# Relations of Ruminal Fermentation Parameters and Microbial Matters to Odd- and Branched-Chain Fatty Acids in Rumen Fluid of Dairy Cows at Different Milk Stages

**DOI:** 10.3390/ani9121019

**Published:** 2019-11-22

**Authors:** Keyuan Liu, Yang Li, Guobin Luo, Hangshu Xin, Yonggen Zhang, Guangyu Li

**Affiliations:** 1 Institute of Special Economic Animal and Plant Science, Chinese Academy of Agricultural Sciences, Changchun 130112, China; liukeyuan0212@163.com; 2 Department of Animal Science and Technology, Northeast Agricultural University, Harbin 150030, China; liyang1405053@sina.com (Y.L.); guobinluo@126.com (G.L.); laura_liuky@foxmail.com (H.X.); 3 Zhejiang NHU Company Ltd., Shaoxing 312500, China

**Keywords:** fermentation parameters, microbial populations, microbial bases, odd- and branched-chain fatty acids, lactation stages

## Abstract

**Simple Summary:**

The objective of this study was to determine the relationships between milk odd- and branched-chain fatty acids (OBCFAs) and ruminal fermentation parameters, microbial populations, and base contents. Significant relationships existed between the concentrations of C11:0, *iso*-C15:0, *anteiso*-C15:0, C15:0, and *anteiso*-C17:0 in rumen and milk. The total OBCFA content in milk was positively related to the acetate molar proportion but negatively correlated with isoacid levels. The adenine/N ratio was negatively related to milk OBCFA content but positively associated with the *iso*-C15:0/*iso*-C17:0 ratio.

**Abstract:**

The purpose of this research was to evaluate whether relationships exist between odd- and branched-chain fatty acids (OBCFAs) originating from milk fat and the corresponding data of ruminal fermentation parameters, microbial populations, and base contents that were used to mark microbial protein in rumen. Nine lactating Holstein dairy cows with similar body weights and parity were selected in this study, and the samples of rumen and milk were collected at the early, middle, and late stages, respectively. The rumen and milk samples were collected over three consecutive days from each cow, and the ruminal and milk OBCFA profiles, ruminal fermentation parameters, bacterial populations, and base contents were measured. The results showed that the concentrations of OBCFAs, with the exception of C11:0 and C15:0, were significantly different between milk and rumen (*p* < 0.05). The concentrations of *anteiso*-fatty acids in milk were higher than those in rumen, and the contents of linear odd-chain fatty acids were higher than those of branched-chain fatty acids in both milk and rumen. Significant relationships that existed between the concentrations of C11:0, *iso*-C15:0, *anteiso*-C15:0, C15:0, and *anteiso*-C17:0 in rumen and milk (*p* < 0.05). The total OBCFA content in milk was positively related to the acetate molar proportion but negatively correlated with isoacid contents (*p* < 0.05). The populations of *Ruminococcus albus*, *R. flavefacients*, and *Eubacterium ruminantium* were significantly related to milk C13:0 contents (*p* < 0.05). The adenine/N ratio was negatively related to milk OBCFA content (*p* < 0.05) but positively associated with the *iso*-C15:0/*iso*-C17:0 ratio (*p* < 0.05). Milk OBCFAs were significantly correlated with ruminal fermentation parameters, ruminal bacterial populations, and base contents. Milk OBCFAs had the potential to predict microbial nitrogen flow, and the prediction equations for ruminal microbial nitrogen flow were established for OBCFAs in dairy milk.

## 1. Introduction

There is growing awareness that milk fat content can respond to physiological and metabolic health situations [1,2]. The milk fatty acid profile is a dynamic pattern influenced by many factors, such as lactational stage, season, and dietary composition [3,4,5]. The microbial processes of the rumen confer the ability to convert feeds into available nutrients for the ruminant animal [6]. Odd- and branched-chain fatty acids (OBCFAs) of ruminant milk generally originate from rumen bacteria [7] and are mainly present in bacterial membrane lipids [8]. Some studies have discussed the potential of OBCFAs as markers of rumen fermentation and ruminal bacteria [9,10,11,12].

Lactating dairy cow digestion is strongly determined by the microbial population in the rumen. In the rumen, microbial fermentation of feedstuffs produces volatile fatty acids (VFAs), which are the main energy supply substances in ruminants. There is a significant relationship between ruminal pH and the profile of VFAs available for absorption [13]. Hence, the composition and amount of milk fatty acids are determined by the proportions and the total amounts of fermentation end-products in ruminants [14]. Many studies have examined the effects of microbial protein synthesis and microbial nucleic acid composition in the rumen on protein nutrition [15]. The composition of the ruminal microbial ecosystem in the forestomach of ruminants is known to be affected by the type and quantity of the ration, feeding intervals, specific additives (e.g., antibiotics), and the host animal itself [16]. To identify the relationships between bacterial populations and milk OBCFA concentrations, seven kinds of bacteria species (cellulolytic or amylolytic bacteria) were selected for the current study. *Ruminococcus albus*, *R. flavefaciens*, *Fibrobacter succinogenes* [17], and *Eubacterium ruminantium* [18] are the predominant ruminal cellulolytic bacteria. The genus *Butyrivibrio fibrisolvens* is a heterogeneous bacterial taxon [19]. *Selenomonas ruminantium* [20] and *Streptococcus bovis* [21] are important for the degradation of starch and lactate, which are abundant in high-grain diets.

However, there are few studies on the relationships between milk OBCFAs during different lactation stages and ruminal bacterial populations. The objective of this research was to estimate the potential use of OBCFAs in milk to predict ruminal fermentation parameters and rumen microbial matter. First, we investigated whether there were relationships between the contents of OBCFA in milk and rumen, and the fermentation parameters, bacterial populations, and bases of milk during different stages of lactation. Second, we developed equations for fermentation parameters, bacterial populations, and bases using the independent datasets of OBCFAs and identified the best OBCFA combination.

## 2. Materials and Methods

### 2.1. Animals and Basal Diets

Nine lactating Holstein dairy cows of similar body weights (650 ± 33 kg body weight) were examined at the same fetal time, and samples of rumen and milk were collected at the early, middle, and late lactation stages. The milk yields were 35.44 ± 2.63, 37.62 ± 2.85, and 26.98 ± 2.79 kg/d in the early, middle, and late stages, respectively. The cows were fed total mixed rations (TMRs), whose composition and nutrition levels are shown in Table 1, according to the dairy nutrient requirements of the NRC (national research council) (2001) [22].

### 2.2. Samples Collection and Analysis Method

All the TMR samples were analyzed for DM, nitrogen (N) (AOAC 968.06), calcium (Ca) (AOAC 927.02), and total phosphorus (TP) (AOAC 965.17) according to the procedures of the AOAC (Association of Official Analytical Chemists) (1990) after air-drying at 60 ± 5 °C [23]. The content of crude protein (CP) was calculated as N × 6.25. The acid detergent fiber (ADF) and neutral detergent fiber (NDF) concentrations were analyzed based on the procedures described by Van Soest et al. [24] using the Ankom system (Ankom 220 fiber analyzer; Ankom, New York, USA) with a heat-stable α-amylase and expressed exclusive of residual ash. Net energy for lactation (NE_L_) at a production level was calculated using an NRC summative approach from the dairy nutrient requirement [22].

The rumen contents were evacuated via the gastric canal over 3 consecutive days. One part of the rumen contents was filtered through four layer of cheesecloth and the filtrates were preserved at −20 °C for the analysis of the concentrations of VFAs and ammonia nitrogen (NH_3_-N), after determining the pH that was obtained from samples by a pH meter (Sartorius Basic pH Meter, Gottingen, Germany). The filtered samples were treated according to the description of Li and Meng [25]. Then, the contents of VFAs were analyzed by a gas chromatography (GC 2010, Shimadzu, Tokyo, Japan) with an FFAP capillary column (HP-INNOWAX, 30 m × 0.25 mm × 0.2 μm, Agilent, California, USA), and using an ammonia-sensing electrode (Expandable Ion Analyzer EA 940, Orion, Massachusetts, USA) to determine the concentration of NH_3_-N.

The other part of the rumen contents were squeezed through two layers of cheesecloth, and about 5 mL was preserved at −80 °C for the extraction of DNA [26] and nearly 100 mL was preserved at −20 °C for the analysis of OBCFA, microbial bases, and the total nitrogen (N). The samples of OBCFA were treated according to the description of Zhang et al. [27] and analyzed by a gas chromatography (GC 2010, Shimadzu, Tokyo, Japan) with an SP-2560^TM^ column for fatty acid methyl esters (100 m × 0.25 mm × 0.2 μm, Supelco, Pennsylvania, USA). The carrier gas was highly pure, and the injector pressure was held constant at 266.9 Kpa. The initial oven temperature was held at 170 °C for 30 min, and increased at 1.5 °C/min to 200 °C and held for 20 min, and then increased by 5 °C/min to 230 °C and held for 5 min. The rumen bases were extracted from freeze-dried samples using perchloric acid, as described by Vlaeminck et al. [9]. The standards of individual bases (≥99.5%, Aladdin, Shanghai, China) were formulated to a concentration of 50 mg/L. The mixed solution mixed by the standard base solutions with the same volume was serially diluted into 5 gradients and subsequently analyzed by HPLC (high performance liquid chromatography) using a C18 column (5 μ, 250 × 4.6 mm, Diamonsil, Guangzhou, China). The buffer solution (20 mM NH_4_H_2_PO_4_) was run isocratically at 1 mL/min, and the effluent was monitored at 254 nm.

DNA was extracted from the rumen contents by the bead-beating procedure described previously by Reilly and Attwood [28]. In detail, 1.5 mL of rumen liquid was placed into a 2-mL centrifuge tube and later centrifuged for 5 min at 12,000 g/min, and the supernatant was subsequently removed. Phosphate buffer saline (PBS; 1.5 mL; pH = 8.0) was then added, and the sample was mixed and centrifuged for 5 min at 12,000 g/min. The supernatant was then removed. CTAB (hexadecyl trimethyl ammonium bromide) buffer (800 μL; sterilized solution containing 4 g of CTAB, 16.364 g of NaCl, 20 mL of 1 mol/L Tris-HCl, pH = 8.0, and 8 mL of 0.5 mol/L EDTA (Ethylene Diamine Tetraacetic Acid) to a final volume of 200 mL) was then added, and the sample was mixed, cultured for 20 min in 70 °C, and centrifuged for 10 min at 10,000 g/min. The supernatant was then transferred to a new centrifuge tube, and 5 μL of RNA enzymes (10 mg/mL) were added. The sample was mixed and then incubated for 30 min at 37 °C. An equal volume of phenol/chloroform/isoamyl alcohol 25:24:1 solution was then added, and the sample was mixed for 15 to 30 s until a white emulsion appeared and then centrifuged for 10 min at 13,000 rpm. The supernatant was added to a new centrifuge tube, and the last step was then repeated until the interface became clear. After a clear interface was obtained, an 0.8-fold volume of the isopropyl alcohol mixture was added into the tube, and the sample was gently mixed, placed at room temperature for 5 min, and then placed at −20 °C overnight. The tube was then centrifuged for 15 to 20 min at 13,000 g/min, and the supernatant was carefully decanted to leave a white DNA precipitation in the bottom of the tube. Ice-cold 70% ethanol (750 μL) was then added to the tube to gently resuspend the DNA precipitation, and the sample was then centrifuged for 10 to 15 min at 12,000 g/min. The supernatant was decanted, and the DNA was allowed to air dry. The DNA was then dissolved with 100 μL (depending on the precipitation volume) of TE (Tris-EDTA) buffer and incubated at 70 °C for 5 min. The DNA solution was then stored at −20 °C until use. The air-dried DNA pellet was redissolved in TE buffer (10 mmol/L Tris-HCl, 1 mmol/L EDTA, pH 8.0) and diluted to concentrations of 10 ng/μL and stored at 4 °C until real-time PCR amplification. The bacterial 16SrRNA genes were amplified using absolute quantification PCR (qPCR). The standard DNA in real-time PCR used plasmid DNA containing the respective target gene sequence, which was obtained by PCR cloning using the species-specific primer set. The specific method was described by Singha et al. [29]. The primer sequences for the 16SrRNA genes and specific amplifications of the correct size are shown in Table 2. The qPCR protocol was performed with ABI 7500 system software (ABI 7500, Massachusetts, USA) using TOYOBO (Osaka, Japan) DNA Master SYBR Green II.

Nearly 100-mL milk samples were collected and preserved at −20 °C, and treated the samples that referred to a description of Vlaeminck et al. [28]. The OBCFA compositions were analyzed by a gas chromatography (GC 2010, Tokyo, Shimadzu, Japan) and the analysis program was the same as the rumen samples.

### 2.3. Statistical Analyses

All data statistical analyses were performed using SAS 9.2 (SAS Institute Inc.,Cary, NC, USA).

The data of variable analysis was done by the MIXED procedure. The MIXED model procedure with the inclusion of the random effect of the study was as described by St-Pierre [32]. The effect of different dietary F:C ratios on OBCFAs were estimated following:

(1) $$Y_{ijk}=\mu +T_{i}+P_{j}+C_{k}+\epsilon_{ijk},$$

where $Y_{ijk}$ is the individual observation, μ is the overall mean, $T_{i}$ is the effect of the dietary treatment (i = 3; F:C = 30:70, 50:50, and 70:30), $P_{j}$ is the effect of the experimental period, $C_{k}$ is the effect of the cow, and $\epsilon_{ijk}$ is the residual error. The effect of the cow was treated as a random effect. For all statistical analyses, significance was declared at *p* < 0.05.

The correlation between the milk OBCFA profile, and rumen OBCFA concentrations, and data, which were obtained from the VFAs, NH_3_-N, and pH, were analyzed by CORR PROC using the Pearson correlation method. The correlations were determined to be significant at *p* ≤ 0.05.

The data of milk OBCFA were considered as an independent data set and the data of VFAs, NH_3_-N, and pH were a dependent dataset. Multiple regression was applied using the STEPWISE method of the REG procedure. The SLENTRY and START values were all 0.05. The equations were determined by least squares estimation (*p* ≤ 0.05). The regression equations were evaluated based on the root mean square error (RMSE) and coefficient of multiple determinations (R^2^) of the regression model:

(2) $$\mathrm{RMSE}=\sqrt{\frac{1}{n}\times \sum_{i=1}^{n}{\left(y_{i}-{\hat{y}}_{i}\right)}^{2}},$$

where n is the number of observations, and $y_{i}$ and ${\hat{y}}_{i}$ are the observed and predicted values, respectively.

## 3. Results

### 3.1. Changes during Different Milk Stages in OBCFA Production in Rumen Fluid and Milk

The changes in OBCFAs in rumen fluid and milk fat are shown in Table 3. The C11:0 and C13:0 contents were significantly higher in the late milk stage than in the early and middle stages, while the concentrations of C15:0 and *anteiso*-C17:0 were lower in the late stage than in the other stages in the rumen fluid (*p* < 0.05). The contents of *anteiso*-C15:0 and *iso*-C16:0 were most abundant in the late milk stage, and *iso*-C17:0 concentrations were lowest in milk fat (*p* < 0.05).

**Figure 1:**
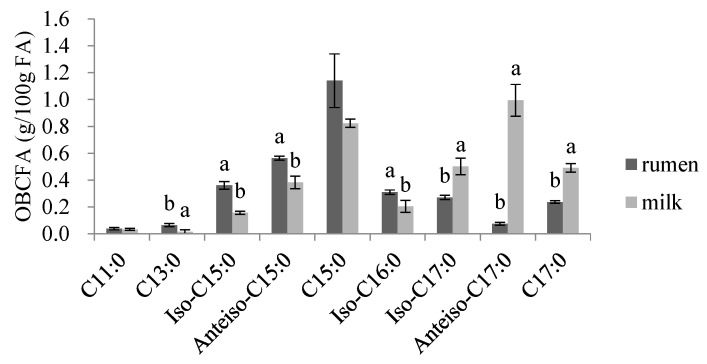
Comparison of rumen fluid and milk OBCFA of different milk stages. The data are means and deviations of three milk stages and error bars the show standard error of the mean. Bars without a common letter (a and b) differ (*p* < 0.05). OBCFA, odd- and branched-chain fatty acids.

The total OBCFA and odd *anteiso*-chain fatty acids contents contained in milk were higher than those in rumen, and the linear odd-chain fatty acids were more abundant both in milk and rumen (Figure 1). Except for C15:0, the concentrations of other OBCFAs were significantly different in milk and rumen. Fatty acids with 15 carbon atoms were the major kinds in rumen, but the *anteiso*-chain fatty acids were much more abundant than other kinds in milk, especially concentrations of *anteiso*-C17:0.

### 3.2. Correlation between Rumen and Milk OBCFA during Different Milk Stages

The contents of OBCFA in rumen fluid were significantly related to that in milk fat (Table 4). Significant relationships of concentrations of C11:0, *iso*-C15:0, *anteiso*-C15:0, C15:0, and *anteiso*-C17:0 were found between rumen fluid and milk fat (*r =* 0.39~0.66, *p* = 0.0002–0.04). The concentrations of C11:0 in milk were significantly related to the concentrations of *anteiso*-C15:0 in rumen (*r =* 0.39, *p* = 0.04). As for the milk, C13:0 contents were significantly correlated with the ruminal *anteiso*-C15:0, C15:0, and total OBCFA concentrations (*r =* 0.48~0.52, *p* = 0.005–0.01). The concentrations of *iso*-C15:0 in milk were positively associated with the contents of C11:0, C13:0, and *anteiso*-C15:0 in rumen fluid (*r =* 0.39~0.41, *p* = 0.03–0.04). The contents of *anteiso*-C15:0 in milk were significantly related to the contents of C11:0, C13:0, and total OBCFA in rumen fluid (*r =* 0.46–0.65, *p* = 0.0002–0.02). The milk C15:0 contents were positively correlated with the concentrations of *anteiso*-C15:0 and total OBCFA in rumen (*r =* 0.58–0.62, *p* = 0.001–0.002). The contents of *iso*-C16:0 in milk were positively correlated with the ruminal C11:0 contents (*r =* 0.44, *p* = 0.02); however, they were negatively related to the ruminal *anteiso*-C17:0 contents (*r =* −0.39, *p* = 0.04). There was significantly relationship between the concentrations of *iso*-C17:0 in milk and the concentrations of C11:0 in rumen (*r =* −0.59, *p* = 0.001). Besides, the contents of *anteiso*-C17:0 in milk were negatively linked with the contents of *iso*-C15:0, C15:0, and total OBCFA in rumen (*r =* −0.39 to −0.49, *p* = 0.01–0.046). A negative relationship that existed between the milk C17:0 contents and ruminal C11:0 contents (*r =* −0.44, *p* = 0.02).

### 3.3. Simple Statistics of Experimental Data Used for Model Development

Simple statistical data were gathered from of the present experimental conditions shown in Table 5. According to the CV%, the *Butyrivibrio flarisolvens* population changed less than the other bacterial populations. There was a wide range of rumen fermentation parameters in this research. With regard to rumen VFAs, larger differences were observed in isoacids than in linear-chain VFAs in the rumen. Compared to the variation of NH_3_-N, the pH was more stable. With regard to the rumen bacterial bases, the variations were similar to each other.

### 3.4. Relationship of Milk OBCFA Pattern during Different Milk Stages to Ruminal Fermentation Parameters, Bacteria Populations, and Microbial Bases

Relationships existed between the fermentation parameters in rumen fluid and the OBCFA patterns (Table 6). Although there no significant correlations that were found between the molar proportions of acetate and individual OBCFA concentrations, the molar proportions of acetate were positively related to total OBCFA concentrations (*r* = 0.40, *p* = 0.04). A negative correlation existed between the propionate molar proportions and *iso*-C17:0 concentrations (*r* = −0.39, *p* = 0.045). The total OBCFA concentrations were negatively correlated with the molar proportions’ isobutytate (*r* = −0.49, *p* = 0.01) while the concentrations of *anteiso*-C15:0 were positively associated with the molar proportions’ butyrate (*r* = 0.47, *p* = 0.01). There was a negative relationship between the milk C15:0 concentration and isovalelate proportion in rumen (*r* = −0.41, *p* = 0.03). The total OBCFA concentrations were negatively related to isovalelate and valelate proportions (*r* = −0.48 to −0.49, *p* = 0.01) but were positively correlated with total VFA contents (*r* = 0.51, *p* = 0.007) and the *iso*-C15:0/*iso*-C17:0 ratio and *anteiso*-C15:0/anteiso-C17:0 ratio (*r* = −0.56 to −0.60, *p* = 0.001–0.002). The concentrations of NH_3_-N were negatively related to *iso*-C15:0 and *anteiso*-C15:0 concentrations (*r* = −0.50 to −0.56, *p* = 0.003–0.008), but positively correlated with *anteiso*-C17:0 concentrations (*r* = 0.47, *p* = 0.01). However, there were no apparent relationships between pH and OBCFA concentrations.

The relationships between bacterial populations and OBCFA concentrations are shown in Table 7. The copy numbers of *Fibrobacter succinogenes* were not related to single OBCFAs concentrations, but positively linked with the *iso*-C15:0/*iso*-C17:0 ratio (*r* = 0.47, *p* = 0.01). The concentrations of C11:0 and C13:0 were positively correlated with the populations of *Ruminococcus albus* (*r* = 0.53–0.57, *p* = 0.002–0.005). The copies of *Ruminococcus flavefaciens* were significantly related to the C13:0 and total OBCFA concentrations (*r* = 0.38–0.50, *p* = 0.009–0.049). A positive correlation was found between C13:0 concentrations and *Eubacterium ruminantium* copy numbers (*r* = 0.43, *p* = 0.03). The ratio of *anteiso*-C15:0 to *anteiso*-C17:0 was positively correlated with *Streptococcus bovis* copies (*r* = 0.48, *p* = 0.01); however, no significant relationships that were found between *Selenomonas ruminantium* copies and individual OBCFA concentrations. The populations of *Selenomonas ruminantium* were positively associated with the total OBCFA and branched-chain fatty acid contents (*r* = 0.39–0.57, *p* = 0.002–0.04).

Several relationships were found between the microbial bases and OBCFA patterns (Table 8). Cytosine concentrations were negatively related to *iso*-C17:0 and total OBCFA concentrations (*r* = −0.39 to −0.50, *p* = 0.007–0.04). The concentrations of uracil were significantly correlated with the *iso*-C17:0 content, the sum of *iso*-C15:0 and *iso*-C17:0 contents (*r* = −0.51 to −0.54, *p* = 0.003–0.004). The concentrations of guanine were negatively related to the concentrations of *iso*-C17:0, the total OBCFA contents, and the sum of *iso*-C15:0 and *iso*-C17:0 contents (*r* = −0.41 to −0.54, *p* = 0.004–0.03). A negative relation was presented by the concentrations of adenine and C17:0 (*r* = −0.39, *p* = 0.04), while the contents of *anteiso*-C15:0 and the C15:0/C17:0 ratio were positively linked with the adenine concentrations (*r* = 0.44–0.49, *p* = 0.01–0.02). The correlations of microbial protein marked by different microbial bases/N ratio with the OBCFAs are also shown in Table 8. The cytosine/N ratio was negatively related to total OBCFA concentrations in milk (*r* = −0.40, *p* = 0.04). In addition, some negative relationships were found between the guanine/N ratio and some milk OBCFAs, which were *iso*-C17:0 and the sum of *iso*-C15:0 and *iso*-C17:0 (*r* = −0.49 to −0.55, *p* = 0.003–0.01). The adenine/N ratio was negatively connected with *iso*-C17:0, *anteiso*-C17:0, the sum of *iso*-C15:0 and *iso*-C17:0, the sum of *anteiso*-C15:0 and *anteiso*-C17:0, the total *iso*-branched chain fatty acids, the total *anteiso*-branched chain fatty acids, and the total branched chain fatty acids in milk (*r* = −0.42 to −0.59, *p* = 0.001–0.03). However, a positive relationship was found between the adenine/N ratio and the ratio of *iso*-C15:0 and *iso*-C17:0 in milk (*r* = 0.50, *p* = 0.01).

The quadratic, ratio and reciprocal of milk OBCFAs were used in the predicted models shown in Table 9, Table 10 and Table 11, respectively. The equations of fermentation parameters, with the exception of that for valerate, were all significant. According to the R^2^ value, the isobutyrate and butyrate models were more accurate than those for other VFAs. The regression equations showed that the ruminal acetate, propionate, and isobutyrate molar proportions and pH had inverse proportional relationships to milk C11:0 concentrations. In addition, inverse proportional relationships existed between the ruminal isobutyrate proportion and milk C17:0 concentrations, between the ruminal butyrate proportion and milk *anteiso*-C15:0 concentrations, and between ruminal NH_3_-N and milk *anteiso*-C15:0 concentrations. The accuracy of the prediction equations for different bacterial populations in the rumen was variable. Regression analysis showed that milk C11:0 concentrations had inverse proportional relationships with *Eubacterium ruminantium*, *Ruminococcus albus*, and *Streptococcus bovis* populations in the rumen. Inverse proportional relationships were found between milk *iso*-C15:0 content and ruminal *Fibrobacter succinogenes* populations as well as between milk C17:0 content and ruminal *Selenomonas ruminantium* populations. Significant regression equations for ruminal microbial markers were obtained using milk OBCFAs as an independent data set. Additionally, the compositions of these equations were complex.

## 4. Discussion

The OBCFA in milk are mainly derived from the ruminal bacterial cell membrane, which was already recognized half a century ago [7]. For reasons previously described by Fievez et al. [33], the milk OBCFA profile was not completely similar to the rumen OBCFA profile. Different proportions of OBCFAs in the rumen and milk were also found in this research. In this study, the fatty acids with 15-carbon atoms were more plentiful than other components in the rumen, but the contents of these fatty acids and *iso*-C16:0 were much lower than those in milk. The summary statistics by Vlaeminck et al. [34] suggested that the linear odd-chain fatty acids accounted for the major proportion of milk OBCFAs. The linear odd-chain fatty acid contents were also much higher in the rumen than in milk in our study. Previous studies have indicated that the mammary gland tissue can de novo synthesize odd-chain fatty acids and their *anteiso*-isomers, through the incorporation of propionyl-CoA instead of acetyl-CoA [35,36,37]. Milk secretion of linear odd-chain fatty acids is higher than that of these fatty acids in duodenal flow, which further demonstrates endogenous chain elongation in the mammary gland [34]. This research also found that the contents of odd-chain fatty acids, except those of C15:0, were higher in milk than in the rumen. Scheerlinck et al. [38] summarized a representative subset of 92 milk samples adopted from a sample database of different dietary treatments, and they reported that the highest concentrations of *iso*-C17:0, *anteiso*-C17:0, and some mono-unsaturated fatty acids were observed in milk. Additionally, Vlaeminck et al. [39] reported that two-carbon elongation of branched-chain FAs occurs postruminal. Increased contents of *anteiso*-C17:0 in milk were found in this research.

At the beginning of lactation, cows are in a negative energy balance, causing the mobilization of adipose fatty acids and the synthesis of these long-chain fatty acids in milk fat [40]. The OBCFAs with 14- and 15-carbon atoms, such as *iso*-C14:0, *iso*-C15:0, C15:0, and *anteiso*-C15:0, were increased in early lactation, which changed with the lactation curves of the short- and medium-chain fatty acids in milk [41]. Increased levels of ruminal C15:0 contents were also found in early lactation in this study. In contrast, levels of OBCFAs with 17-carbon atoms decreased during early lactation, which showed a similar pattern to that of long-chain fatty acids in milk [41]. In the present study, however, the concentrations of *iso*-C17:0 in milk were more abundant in early lactation, which may be caused by the synthesis of these fatty acids. A previous study found that long-chain fatty acids are de novo synthesized from some fatty acids, which causes rumen acidosis in milk at an early lactation stage [40].

In the mammary gland, fatty acids with 4- to 14-carbon atoms mainly come from endogenous synthesis, and the long-chain fatty acids greater than 16-carbon atoms are mostly derived from exogenous transformation [42]. In addition, Verbeke et al. [43] found that the mammary gland had the ability to synthesize fatty acids from 2-methylbutyryl-CoA, isovaleryl-CoA, and isobutyryl-CoA. The same types of fatty acids with less than 16-carbon atoms collected from rumen fluid and milk were significantly or nearly related to each other in this research. These results may be due to the minor amount of fatty acids synthesized in the mammary gland. Odd-chain fatty acids are further metabolized under the action of dehydrogenase in the mammary gland, but the transformation of C17:0 to C17:1 was statistically significant [44]. Hence, negative correlations existed between the milk fatty acids with 17-carbon atoms and other ruminal fatty acids in this study. The C15:0_milk_/C15:0_duodenum_ and C17:0 + C17:1_milk_/C17:0_duodenum_ values changed from 1.5 to 2.25 [34,45,46], which suggested that the metabolic and transport processes of the two types of fatty acids were different in the mammary gland.

Increasing the acetate supply has a positive effect on milk yield and milk fat content [47]. In this study, a positive correlation existed between acetate content in the rumen and the total concentrations of OBCFAs in milk. Vlaeminck et al. [10] found that milk *iso*-C14:0 and *iso*-C15:0 contents are positively correlated with ruminal proportions of acetate. We also found a positive relationship between the concentrations of *iso*-C17:0 in milk and the molar proportions of ruminal acetate, but this relationship was not significant. Moreover, milk *iso*-C14:0 and *iso*-C15:0 contents are negatively related to ruminal propionate proportions according to Vlaeminck et al. [10]. In this work, the contents of ruminal propionate were negatively related to *iso*-fatty acids, especially milk *iso*-C17:0. Negative relationships were found between ruminal *iso*-fatty acids and the contents of total OBCFAs in milk. Isoacids are also used as primers for branched-chain fatty acids [34], and their contents are low in the rumen [48,49]. Compared to linear and *iso*-fatty acids, *anteiso*-fatty acids are more susceptible to effects on bacterial membrane properties [50]. Cabrita et al. [51] found a negative correlation between the concentrations of ruminal NH_3_ and milk *anteiso*-C17:0. In this study, there was a negative relationship between NH_3_-N concentrations in the rumen and *anteiso*-C15:0 concentrations in milk. However, milk *anteiso*-C17:0 concentrations were significantly positively related to ruminal NH_3_ concentrations. In addition, the ruminal NH_3_-N concentrations were positively related to *iso*-C15:0/*iso*-C17:0 or *anteiso*-C15:0/*anteiso*-C17:0 values.

Variation in the OBCFA profile leaving the rumen has been suggested to reflect changes in the relative abundance of specific ruminal bacterial populations [33]. Any deviation in the rumen’s inner circumstance may influence the microbial population and its fermentation products [52]. Vlaeminck et al. [34] summarized that *Fibrobacter succinogenes* abundantly produces linear odd-chain fatty acids and fatty acids with 15-carbon atoms. In the present study, *Fibrobacter succinogenes* populations in the rumen were not significantly related to separate OBCFA concentrations, but they were positively correlated with the *iso*-C15:0/*iso*-C17:0 ratio in milk. Hence, the different results from the cultivable ruminal bacteria might be due to de novo fatty acid synthesis and elongation in the mammary gland. Some previous studies have reported that cellulolytic bacteria contain a high amount of *iso*-fatty acids, with differences between different species [19,53,54,55]. Specifically, *Ruminococcus flavefaciens* has a high level of odd-chain *iso*-fatty acids, and *Ruminococcus albus* has enriched even-chain *iso*-fatty acids [34]. Although the contribution of OBCFA endogenous synthesis in milk from dairy cows was negligible [56], linear odd-chain fatty acids, or their *anteiso*-isomers, can be de novo synthesized in the mammary gland [35]. Ifkovits and Ragheb [53] found that a pure culture of *Eubacterium ruminantium* contains high levels of C15:0 and *iso*-C15:0, but Minato et al. [54] reported that *Eubacterium ruminantium* is enriched in *anteiso*-C13:0. These studies suggest that different isolated strains of *Eubacterium ruminantium* have variable OBCFA profiles. In the present work, *Ruminococcus albus*, *Ruminococcus flavefaciens*, and *Eubacterium ruminantium* abundance were correlated with C13:0 contents in milk, which suggested that milk C13:0 might reflect the population of cellulolytic bacteria. The copy number of *Fibrobacter succinogenes* was positively correlated with the ratio of *iso*-C15:0 to *iso*-C17:0, which indicated that the transformation of *iso*-C15:0 to *iso*-C17:0 may occur in *Fibrobacter succinogenes*. Various bacterial strains of *Butyrivibrio fibrisolvens* form a heterogeneous group of bacteria that ferment a wide array of substrates [19], including fiber, starch, and fatty acids [52], and produce large amounts of lactate and acetate [57], which can increase both the growth rate [58] and butyrate production rate [59]. These previous studies showed that *Butyrivibrio fibrisolvens* is a key link between long-chain and short-chain fatty acids, which may be attributed to the relationship between the ruminal microbial population and milk OBCFA concentrations. Previous studies have suggested that amylolytic bacteria are relatively enriched in linear odd-chain fatty acids [34]. The populations of *Selenomonas ruminantium* in the rumen are highly correlated with total OBCFAs and branched-chain fatty acid concentrations in milk, suggesting that OBCFA concentrations in milk are involved in a complex interaction with ruminal microorganisms.

Vlaeminck et al. [9] first suggested that milk OBCFAs can be used as markers for the duodenal flow of microbial matter and found that changes in OBCFAs are closely associated with changes in uracil and purine bases in the rumen, thus confirming the potential of OBCFAs to predict microbial matter in the rumen. Several significant correlations existed between ruminal microbial base concentrations and milk OBCFA contents in the present study and in previous studies. Cabrita et al. [51] found that milk C17:0, *iso*-C17:0, and *anteiso*-C17:0 contents are significantly and negatively associated with dietary crude protein content, and they suggested that C17:0 is a marker of protein deficiency. This study implied that fatty acids with 17-carbon atoms in milk are associated with protein degradability in the rumen. Additionally, protein degradability is one of the factors that may affect rumen microbial growth [60]. The odd-chain fatty acid synthesis process is different from that of branched-chain fatty acids. [34]. Wongtangtintharn et al. [61] showed that all branched-chain fatty acids equally inhibit fatty acid synthesis. In this study, changes in the relationships between milk branched-chain fatty acids and ruminal microbial base contents were found. Moreover, the elongation of *iso*-C15:0 and *anteiso*-C15:0 adds to the existing *iso*-C17:0 and *anteiso*-C17:0 content [33], which may explain the lower *iso*-C15:0/*iso*-C17:0 and *anteiso*-C15:0/*anteiso*-C17:0 ratios in milk compared to the ratios found in rumen bacteria or duodenal content. The *iso*-C15:0/*iso*-C17:0 ratio in milk was positively correlated with the adenine/N ratio in the rumen, which suggested that the *iso*-C15:0/*iso*-C17:0 ratio in milk reflects the abundance of ruminal microbial.

Vlaeminck et al. [10] developed equations based on milk OBCFAs to predict molar proportions of individual VFAs in the rumen. Bhagwat et al. [12] improved the prediction accuracy of VFA proportions from measured milk OBCFA concentrations through the development and application of quadratic terms and interactions as well as the ratio of linear expressions. Bhagwat et al. [12] indicated that the more complex methods provide better predictions with fewer OBCFAs. Hence, we established an equation with mixed variables containing the first and second degree of dependent data as well as the ratio of any two datasets and the reciprocal of each dependent datasets in this research. Using this method, we generated a prediction model for major bacterial populations and microbial protein markers. In this model, there were some inversely proportional and quadratic relationships from the predicted equations, which suggested that there were some nonlinear relationships between milk OBCFAs and ruminal fermentation parameters, ruminal bacterial populations, and base contents.

## 5. Conclusions

OBCFAs originating from milk were significantly correlated with ruminal fermentation parameters, ruminal bacterial populations, and base contents. The results suggested that milk odd-chain fatty acids have the potential to be used as a noninvasive technique to assess rumen function in terms of microbial populations, substrates, and interactions. To increase the accuracy of the predicted equations for ruminal parameters, ruminal bacterial populations, and base contents established based on milk OBCFAs, a large number of experiments are required.

## Figures and Tables

**Table 1:** Feed ingredients and chemical composition (g/kg DM) of the rations.

Ingredients	Content
Alfalfa hay	73
Chinese wildrye	43
Corn silage	334
Corn	220
Soybean meal	41
DDGS	214
Cottonseed meal	58
Molasses	5.0
NaCl	3.0
CaHPO_4_	1.6
Limestone	5.4
Premix *	3.0
Nutrient levels	
NEL(MJ/kg) ^†^	8.71
CP	162
NDF	312
ADF	183
Ca	8.7
TP	4.6

* The premix provided the following per kg of diets: Vitamin A 330,000 IU, Vitamin D 60,000 IU, Vitamin E 1000 IU, Zn 2100 mg, Mn 1500 mg, Cu 535 mg, Se 12 mg, I 45 mg. ^†^ NEL was an estimated value [22]. DM, dry matter; DDGS, Corn distillers dried grains; CP, crude protein; NDF, neutral detergent fiber; ADF, acid detergent fiber; TP, total phosphorus.

**Table 2:** Primers for real time-PCR.

Bacteria species	Primer Sequence (5′-3′)	Product Size (bp)	Reference
*Fibrobacter succinogenes*	F-GGCGGGATTGAATGTACCTTGAGA	204	Yang (2007) [30]
	R-TCCGCCTGCCCCTGAACTATC		
*Ruminococcus albus*	F-GTTTTAGGATTGTAAACCTCTGTCTT	273	Yang (2007) [30]
	R-CCTAATATCTACGCATTTCACCGC		
*Ruminococcus flavefaciens*	F-GATGCCGCGTGGAGGAAGAAG	278	Yang (2007) [30]
	R-CATTTCACCGCTACACCAGGAA		
*Butyrivibrio fibrisolvens*	F-TAACATGAGTTTGATCCTGGCTC	113	Yang (2007) [30]
	R-CGTTACTCACCCGTCCGC		
*Eubacterium ruminantium*	F-CTCCCGAGACTGAGGAAGCTTG	184	Stevenson, et al. (2007) [31]
	R-GTCCATCTCACACCACCGGA		
*Streptococcus bovis*	F-TTCCTAGAGATAGGAAGTTTCTTCGG	127	Stevenson, et al. (2007) [31]
	R-ATGATGGCAACTAACAATAGGGGT		
*Selenomonas ruminantium*	F-CAATAAGCATTCCGCCTGGG	138	Stevenson, et al. (2007) [31]
	R-TTCACTCAATGTCAAGCCCTGG		

**Table 3:** Changes of different milk stages on OBCFA production in rumen fluid and milk (g/100 g fatty acids).

OBCFA Profile	Rumen Fluid					Milk				
	Early Stage	Middle Stage	Late Stage	SEM	*p*	Early Stage	Middle Stage	Late Stage	SEM	*p*
C11:0	0.03 ^b^	0.04 ^b^	0.05 ^a^	0.003	0.001	0.03	0.03	0.04	0.01	0.17
C13:0	0.06 ^b^	0.06 ^b^	0.08 ^a^	0.004	0.004	0.01 ^b^	0.02 ^a^	0.01 ^b^	0.002	0.04
*Iso*-C15:0	0.39	0.33	0.36	0.04	0.61	0.14	0.15	0.15	0.01	0.12
*Anteiso*-C15:0	0.58	0.55	0.56	0.04	0.79	0.38	0.39	0.38	0.03	0.93
C15:0	1.37 ^a^	1.01 ^b^	1.04 ^b^	0.08	0.004	0.84	0.84	0.78	0.04	0.44
*Iso*-C16:0	0.30	0.30	0.33	0.02	0.62	0.25 ^a^	0.19 ^b^	0.16 ^b^	0.02	0.02
*Iso*-C17:0	0.27	0.26	0.29	0.02	0.33	0.44 ^b^	0.52 ^a^	0.55 ^a^	0.03	0.04
*Anteiso*-C17:0	0.07 ^b^	0.09 ^a^	0.07 ^b^	0.004	0.002	0.83 ^b^	1.13 ^a^	1.01 ^b^	0.07	0.02
C17:0	0.25	0.24	0.23	0.02	0.51	0.57	0.43	0.47	0.06	0.23
TOBCFA	3.32	2.87	3.00	0.14	0.08	3.54	3.57	3.69	0.09	0.42

OBCFA, odd- and branched-chain fatty acids; TOBCFA, the total odd- and branched-chain fatty acids. a, b, c, means with different letters within the same line and the same item differ significantly (*p* < 0.05). SEM = standard error of mean.

**Table 4:** Correlation between rumen and milk OBCFA of different milk stages.

Rumen Fluid	Milk Fat																			
	C11:0		C13:0		*Iso*-C15:0		*Anteiso*-C15:0		C15:0		*Iso*-C16:0		*Iso*-C17:0		*Anteiso*-C17:0		C17:0		TOBCFA	
	*r*	*p*	*r*	*p*	*r*	*p*	*r*	*p*	*r*	*p*	*r*	*p*	*r*	*p*	*r*	*p*	*r*	*p*	*r*	*p*
C11:0	0.40	0.04	0.13	0.53	0.39	0.04	0.50	0.01	0.16	0.43	0.44	0.02	−0.59	0.001	−0.32	0.11	−0.44	0.02	−0.03	0.87
C13:0	0.26	0.19	0.14	0.47	0.41	0.04	0.65	0.0002	−0.01	0.96	0.37	0.06	−0.36	0.07	−0.25	0.21	−0.23	0.25	0.03	0.89
*Iso*-C15:0	−0.02	0.93	0.34	0.08	0.42	0.03	0.25	0.20	0.36	0.07	−0.06	0.76	−0.15	0.46	−0.39	0.046	0.16	0.43	−0.03	0.88
*Anteiso*-C15:0	0.39	0.04	0.48	0.01	0.43	0.03	0.60	0.001	0.58	0.002	−0.24	0.23	−0.004	0.98	−0.30	0.13	0.09	0.65	0.31	0.11
C15:0	0.20	0.33	0.52	0.005	0.20	0.31	0.12	0.54	0.66	0.0002	−0.14	0.50	0.13	0.51	−0.44	0.02	0.22	0.27	0.06	0.76
*Iso*-C16:0	0.08	0.68	−0.07	0.72	0.17	0.39	0.18	0.38	−0.03	0.90	0.38	0.052	−0.19	0.34	−0.14	0.48	−0.05	0.81	−0.02	0.90
*Iso*-C17:0	0.29	0.14	0.18	0.36	0.27	0.18	0.37	0.06	0.45	0.02	−0.07	0.74	0.003	0.99	−0.22	0.26	−0.14	0.47	0.13	0.52
*Anteiso*-C17:0	−0.09	0.65	−0.12	0.57	−0.13	0.52	0.06	0.78	−0.02	0.93	−0.39	0.04	0.31	0.12	0.39	0.04	0.14	0.49	0.34	0.08
C17:0	−0.04	0.83	0.31	0.12	0.02	0.90	−0.03	0.89	−0.01	0.96	−0.29	0.15	0.07	0.72	−0.16	0.44	0.17	0.40	−0.16	0.43
TOBCFA	0.29	0.14	0.49	0.01	0.36	0.07	0.46	0.02	0.62	0.001	−0.06	0.77	−0.07	0.73	−0.49	0.01	0.11	0.57	0.09	0.64

OBCFA, odd- and branched-chain fatty acids; TOBCFA, the total odd- and branched-chain fatty acids. *r* = correlation coefficient.

**Table 5:** Simple statistics of experimental data used for model development.

Variables	N	Mean	SD	Minimum	Maximum	CV%
Milk odd and branched-chain fatty acids (g/100 g fatty acids)						
C11:0	27	0.03	0.02	0.01	0.08	50.53
C13:0	27	0.02	0.01	0.01	0.04	46.25
*Iso*-C15:0	27	0.16	0.03	0.09	0.21	18.39
*Anteiso*-C15:0	27	0.38	0.07	0.23	0.58	19.33
C15:0	27	0.82	0.12	0.64	1.02	14.26
*Iso*-C16:0	27	0.20	0.07	0.10	0.37	34.76
*Iso*-C17:0	27	0.50	0.10	0.23	0.62	19.46
*Anteiso*-C17:0	27	0.99	0.23	0.51	1.44	23.35
C17:0	27	0.49	0.18	0.34	1.08	36.52
TOBCFA	27	3.61	0.25	3.01	4.03	6.94
Rumen fermentation parameters						
Acetate (mmol/mol)	27	523.81	25.31	474.55	577.33	4.83
Propionate (mmol/mol)	27	294.02	18.24	255.62	327.79	6.21
Isobutyrate (mmol/mol)	27	14.84	2.20	10.06	19.35	14.81
Butyrate (mmol/mol)	27	120.65	4.81	112.00	127.81	3.99
Isovalerate (mmol/mol)	27	20.84	2.37	15.89	25.26	11.36
Valerate (mmol/mol)	27	25.84	2.32	21.25	30.63	8.98
TVFA (mmol/l)	27	63.12	13.50	48.76	110.34	21.39
NH3-N (mmol/l)	27	11.69	3.67	4.84	19.95	31.39
pH	27	6.35	0.24	5.87	6.70	3.77
Ruminal bacterial populations (log_10_ copies/mL)						
*Fibrobacter succinogenes*	27	7.99	0.32	7.33	8.56	4.00
*Ruminococcus albus*	27	7.03	0.30	6.55	7.55	4.29
*Ruminococcus flavafaciens*	27	7.73	0.14	7.42	8.02	1.85
*Butyrivibro flarisolvens*	27	8.91	0.07	8.79	9.08	0.80
*Eubacterium ruminantium*	27	6.62	0.28	5.88	7.23	4.19
*Streptococcus bovis*	27	5.27	0.30	4.45	5.74	5.61
*Selenomonas ruminantium*	27	7.35	0.19	6.97	7.62	2.61
Ruminal bacterial bases (g/kg DM)						
Cytosine	27	1.21	0.11	0.98	1.38	8.94
Uracil	27	0.98	0.14	0.63	1.16	14.10
Guanine	27	2.37	0.29	1.60	2.80	12.20
Adenine	27	1.86	0.16	1.43	2.16	8.57
Ruminal bacterial bases/N (g/100 g N)						
Cytosine/N	27	3.11	0.24	2.75	3.66	0.08
Uracil/N	27	2.52	0.33	1.80	3.01	0.13
Guanine/N	27	6.09	0.62	4.52	7.16	0.10
Adenine/N	27	4.80	0.44	4.13	6.11	0.09

TVFA, the total contents of acetate, propionate, isobutyrate, butyrate, isovalerate, and valerate in rumen. OBCFA, odd- and branched-chain fatty acids; TOBCFA, the total odd- and branched-chain fatty acids. SD = standard deviation; CV = coefficient of variation.

**Table 6:** Correlation between ruminal fermentation parameters and milk OBCFA pattern.

Item	Acetate		Propionate		Isobutyrate		Butyrate		Isovalerate		Valerate		TVFA		NH_3_-N		pH	
	*r*	*p*	*r*	*p*	*r*	*p*	*r*	*p*	*r*	*p*	*r*	*p*	*r*	*p*	*r*	*p*	*r*	*p*
C11:0	−0.22	0.28	0.12	0.56	0.36	0.06	0.17	0.41	0.19	0.33	0.26	0.18	−0.31	0.11	0.06	0.75	0.22	0.26
C13:0	0.14	0.48	−0.22	0.28	0.08	0.69	−0.21	0.30	0.16	0.44	−0.05	0.82	−0.20	0.33	0.19	0.34	0.29	0.14
*Iso*-C15:0	−0.07	0.73	0.19	0.35	−0.08	0.69	0.05	0.79	−0.10	0.63	−0.07	0.74	0.18	0.37	−0.50	0.008	−0.05	0.81
*Anteiso*-C15:0	−0.34	0.08	0.31	0.11	0.32	0.11	0.47	0.01	0.11	0.60	0.28	0.16	−0.22	0.27	−0.56	0.003	0.21	0.29
C15:0	0.18	0.37	−0.11	0.60	−0.31	0.11	−0.05	0.81	−0.41	0.03	−0.33	0.097	0.37	0.06	−0.28	0.16	0.20	0.32
*Iso*-C16:0	−0.12	0.54	0.20	0.32	0.19	0.35	0.03	0.90	0.02	0.93	0.22	0.28	−0.06	0.76	−0.21	0.29	−0.31	0.12
*Iso*-C17:0	0.36	0.06	−0.39	0.045	−0.37	0.06	−0.24	0.22	−0.25	0.20	−0.35	0.08	0.11	0.59	0.22	0.27	0.12	0.56
*Anteiso*-C17:0	0.26	0.19	−0.35	0.07	−0.19	0.35	−0.12	0.55	−0.17	0.39	−0.21	0.28	0.17	0.40	0.47	0.01	0.18	0.37
C17:0	0.21	0.28	−0.06	0.77	−0.37	0.06	−0.32	0.0996	−0.28	0.16	−0.38	0.05	0.23	0.26	0.12	0.55	−0.05	0.81
TOBCFA	0.40	0.04	−0.33	0.09	−0.49	0.01	−0.26	0.18	−0.48	0.01	−0.49	0.01	0.51	0.007	0.06	0.76	0.08	0.71
C15:0 + C17:0	0.12	0.57	0.03	0.89	−0.29	0.14	−0.11	0.58	−0.37	0.06	−0.32	0.10	0.37	0.06	−0.25	0.22	0.07	0.74
*Iso*-C15:0 + *iso*-C17:0	0.30	0.13	−0.36	0.06	−0.32	0.10	−0.12	0.55	−0.23	0.26	−0.27	0.17	0.23	0.26	0.02	0.90	0.17	0.39
*Anteiso*-C15:0 + *anteiso*-C17:0	0.14	0.48	−0.25	0.21	−0.07	0.72	0.04	0.83	−0.11	0.58	−0.09	0.65	0.09	0.65	0.30	0.12	0.22	0.27
Total *iso*-fatty acids	0.32	0.0997	−0.34	0.08	−0.28	0.16	−0.24	0.23	−0.18	0.38	−0.19	0.35	0.22	0.27	−0.02	0.94	−0.23	0.24
Total *anteiso*-fatty acids	0.14	0.48	−0.25	0.21	−0.07	0.71	0.05	0.82	−0.11	0.57	−0.09	0.64	0.09	0.64	0.31	0.12	0.22	0.27
Total odd-chain fatty acids	0.09	0.67	0.05	0.81	−0.26	0.19	−0.09	0.65	−0.33	0.096	−0.30	0.13	0.34	0.08	−0.26	0.20	0.10	0.63
Total branched-chian fatty acids	0.16	0.44	−0.25	0.22	−0.08	0.68	−0.03	0.90	−0.19	0.35	−0.09	0.66	0.12	0.55	0.22	0.28	0.15	0.47
C15:0/C17:0	−0.13	0.51	0.04	0.85	0.17	0.40	0.30	0.13	−0.03	0.90	0.18	0.37	−0.04	0.83	−0.31	0.11	0.27	0.17
*Iso*-C15:0/*iso*-C17:0	−0.25	0.21	0.35	0.08	0.14	0.49	0.22	0.28	0.05	0.80	0.16	0.42	0.09	0.65	−0.56	0.002	−0.13	0.52
*Anteiso*-C15:0/*anteiso*-C17:0	−0.32	0.10	0.36	0.06	0.31	0.12	0.30	0.12	0.14	0.50	0.30	0.13	0.07	0.74	−0.60	0.001	0.04	0.84

TVFA, the total contents of acetate, propionate, isobutyrate, butyrate, isovalerate, and valerate in rumen. OBCFA, odd- and branched-chain fatty acids; TOBCFA, the total odd- and branched-chain fatty acids. *r* = correlation coefficient.

**Table 7:** Correlation between bacteria populations and milk OBCFA pattern.

Item	*Fibrobacter succinogenes*		*Ruminococcus albus*		*Ruminococcus flavafaciens*		*Butyrivibro flarisolvens*		*Eubacterium ruminantium*		*Streptococcus bovis*		*Selenomonas ruminantium*	
	*r*	*p*	*r*	*p*	*r*	*p*	*r*	*p*	*r*	*p*	*r*	*p*	*r*	*p*
C11:0	0.17	0.38	0.57	0.002	0.18	0.37	0.12	0.54	0.36	0.06	0.19	0.34	0.01	0.95
C13:0	0.11	0.60	0.53	0.005	0.50	0.009	0.15	0.45	0.43	0.03	0.22	0.27	−0.01	0.97
*Iso*-C15:0	0.37	0.055	−0.03	0.87	0.35	0.07	−0.09	0.65	0.05	0.79	0.02	0.94	−0.05	0.79
*Anteiso*-C15:0	0.18	0.37	0.35	0.07	−0.04	0.83	0.14	0.47	0.22	0.26	0.33	0.09	−0.25	0.20
C15:0	0.21	0.29	0.25	0.21	0.17	0.39	−0.28	0.16	0.05	0.81	0.22	0.28	0.27	0.18
*Iso*-C16:0	0.14	0.48	−0.17	0.39	−0.18	0.36	0.14	0.48	−0.07	0.71	−0.21	0.29	−0.09	0.65
*Iso*-C17:0	−0.25	0.21	−0.03	0.88	0.13	0.53	0.05	0.81	−0.01	0.94	−0.02	0.92	0.33	0.09
*Anteiso*-C17:0	−0.14	0.49	0.01	0.98	0.13	0.51	−0.19	0.34	0.03	0.88	−0.34	0.09	0.36	0.06
C17:0	−0.22	0.27	−0.19	0.34	0.22	0.28	0.18	0.38	−0.09	0.65	−0.01	0.96	0.37	0.06
TOBCFA	0.05	0.81	0.03	0.89	0.38	0.049	−0.03	0.89	0.17	0.40	−0.18	0.38	0.57	0.002
C15:0 + C17:0	0.16	0.42	0.06	0.77	0.16	0.44	−0.04	0.83	−0.03	0.90	0.24	0.23	0.32	0.098
*Iso*-C15:0 + *iso*-C17:0	−0.06	0.77	0.13	0.53	0.28	0.16	0.01	0.95	0.11	0.59	0.09	0.67	0.38	0.051
*Anteiso*-C15:0 + *anteiso*-C17:0	−0.005	0.98	0.09	0.66	0.17	0.39	−0.22	0.27	0.06	0.78	−0.30	0.13	0.27	0.18
Total *iso*-fatty acids	0.02	0.93	−0.03	0.89	0.23	0.25	0.13	0.52	0.06	0.76	−0.10	0.61	0.31	0.12
Total *anteiso*-fatty acids	−0.01	0.96	0.08	0.69	0.16	0.41	−0.22	0.27	0.05	0.79	−0.30	0.13	0.26	0.18
Total odd-chain fatty acids	0.18	0.38	0.11	0.59	0.19	0.35	−0.04	0.84	0.01	0.97	0.24	0.22	0.31	0.12
Total branched-chian fatty acids	0.04	0.86	0.11	0.59	0.18	0.37	−0.15	0.45	0.08	0.69	−0.28	0.16	0.39	0.04
C15:0/C17:0	0.32	0.104	0.33	0.09	−0.05	0.81	−0.32	0.11	0.10	0.63	0.17	0.41	−0.10	0.61
*Iso*-C15:0/*iso*-C17:0	0.47	0.01	−0.05	0.82	0.13	0.51	−0.10	0.63	−0.04	0.86	0.07	0.75	−0.21	0.28
*Anteiso*-C15:0/*anteiso*-C17:0	0.23	0.24	0.29	0.15	−0.12	0.54	0.21	0.28	0.17	0.39	0.48	0.01	−0.31	0.12

OBCFA, odd- and branched-chain fatty acids; TOBCFA, the total odd- and branched-chain fatty acids. *r* = correlation coefficient.

**Table 8:** Correlation between microbial protein marked by nucleic acids, marker:N ratio in rumen (when using nucleic acids as the microbial marker), and milk OBCFA pattern (*n* = 27).

Item	Cytosine		Uracil		Guanine		Adenine		Cytosine/N		Uracil/N		Guanine/N		Adenine/N	
	*r*	*p*	*r*	*p*	*r*	*p*	*r*	*p*	*r*	*p*	*r*	*p*	*r*	*p*	*r*	*p*
C11:0	0.05	0.81	0.20	0.33	0.07	0.74	0.16	0.42	0.02	0.91	0.10	0.62	0.19	0.35	0.08	0.68
C13:0	0.12	0.54	−0.09	0.65	0.13	0.51	0.21	0.28	0.27	0.17	0.00	0.99	−0.23	0.24	0.13	0.50
*Iso*-C15:0	−0.11	0.58	−0.18	0.36	−0.06	0.78	0.18	0.36	−0.13	0.52	−0.05	0.79	−0.14	0.48	0.20	0.32
*Anteiso*-C15:0	−0.06	0.76	0.12	0.56	0.07	0.74	0.49	0.01	−0.07	0.72	−0.17	0.39	0.16	0.44	0.08	0.68
C15:0	−0.19	0.35	−0.32	0.11	−0.28	0.16	0.30	0.13	−0.25	0.21	0.04	0.86	−0.14	0.48	0.10	0.63
*Iso*-C16:0	0.09	0.67	0.38	0.051	0.31	0.12	−0.09	0.66	0.35	0.07	−0.07	0.72	0.26	0.19	0.20	0.31
*Iso*-C17:0	−0.39	0.04	−0.51	0.006	−0.54	0.004	−0.21	0.29	−0.17	0.40	−0.24	0.22	−0.49	0.01	−0.59	0.001
*Anteiso*-C17:0	−0.18	0.36	−0.12	0.55	−0.25	0.20	−0.13	0.52	−0.22	0.27	−0.09	0.65	−0.17	0.40	−0.47	0.01
C17:0	−0.30	0.13	0.11	0.59	−0.23	0.24	−0.39	0.04	−0.12	0.54	−0.13	0.51	0.19	0.35	0.20	0.32
TOBCFA	−0.50	0.007	−0.28	0.16	−0.41	0.03	0.03	0.90	−0.40	0.04	−0.33	0.09	−0.17	0.40	−0.37	0.06
C15:0 + C17:0	−0.28	0.16	−0.04	0.84	−0.19	0.34	0.00	0.99	−0.24	0.23	−0.09	0.65	0.08	0.69	0.22	0.28
*Iso*-C15:0 + *iso*-C17:0	−0.35	0.08	−0.54	0.003	−0.53	0.005	−0.09	0.66	−0.21	0.28	−0.27	0.17	−0.55	0.003	−0.55	0.003
*Anteiso*-C15:0 + *anteiso*-C17:0	−0.18	0.38	−0.04	0.83	−0.21	0.28	−0.001	1.00	−0.26	0.19	−0.16	0.42	−0.13	0.52	−0.49	0.01
Total *iso*-fatty acids	−0.28	0.16	−0.33	0.09	−0.31	0.11	−0.17	0.39	0.05	0.81	−0.34	0.08	−0.37	0.06	−0.42	0.03
Total *anteiso*-fatty acids	−0.18	0.37	−0.05	0.82	−0.22	0.27	−0.01	0.97	−0.26	0.19	−0.16	0.42	−0.13	0.52	−0.49	0.01
Total odd-chain fatty acids	−0.24	0.23	−0.03	0.89	−0.15	0.45	0.04	0.83	−0.23	0.24	−0.08	0.67	0.09	0.66	0.23	0.24
Total branched-chian fatty acids	−0.23	0.26	−0.11	0.60	−0.26	0.19	−0.04	0.86	−0.21	0.30	−0.26	0.19	−0.24	0.22	−0.56	0.002
C15:0/C17:0	0.16	0.42	0.00	0.99	−0.05	0.81	0.44	0.02	0.01	0.96	0.23	0.24	−0.03	0.87	0.06	0.78
*Iso*-C15:0/*iso*-C17:0	0.10	0.60	0.23	0.25	0.23	0.25	0.21	0.28	0.04	0.83	0.10	0.61	0.25	0.21	0.50	0.01
*Anteiso*-C15:0/*anteiso*-C17:0	0.06	0.77	0.22	0.27	0.17	0.39	0.38	0.053	0.02	0.91	−0.05	0.79	0.24	0.22	0.34	0.08

OBCFA, odd- and branched-chain fatty acids; TOBCFA, the total odd- and branched-chain fatty acids. *r* = correlation coefficient.

**Table 9:** Predicted equations of ruminal fermentation parameters from milk OBCFA.

Variables	Predicted Equation	RMSE	R^2^	*P*
Acetate (mmol/mol)	Y=55.73−1.60/(C11:0)	2.38	0.15	0.049
Propionate (mmol/mol)	Y=26.99+1.15/(C11:0)	1.75	0.12	0.0496
Isobutyrate (mmol/mol)	Y=−0.32+0.48/(C17:0)+20.23×C11:0×anteiso−C15:0+1.04/(C11:0)	0.17	0.46	0.002
Butyrate (mmol/mol)	Y=14.37−0.37/(anteiso−C15:0)−4.08×C11:0×C17:0−102.26×C13:0×iso−C16:0	0.41	0.35	0.02
Isovalerate (mmol/mol)	Y=1.13−0.88×C15:0−0.68×anteiso−C17:0×C17:0	0.21	0.28	0.02
Valerate (mmol/mol)	Y=3.21−1.36×C11:0×C17:0−0.38×C15:0×anteiso−C17:0	0.21	0.27	0.08
TVFA (mmol/L)	Y=24.56+155.95×C11:0×C15:0+38.78×anteiso−C17:0×C17:0	12.03	0.27	0.02
NH3-N (mmol/L)	Y=5.46+0.11/(iso−C16:0)+3.71×C11:0×iso−C16:0	2.95	0.40	0.002
pH	Y=6.03+0.15/(C11:0)	0.16	0.22	0.04

R^2^ = coefficient of determination; RMSE = root mean square error. TVFA, the total contents of acetate, propionate, isobutyrate, butyrate, isovalerate, and valerate in rumen.

**Table 10:** Predicted equations of ruminal bacterial populations (log_10_ copies/mL) from milk OBCFA.

Variables	Predicted Equation	RMSE	R^2^	*P*
*Fibrobacter succinogenes*	Y=8.48−0.15×(iso−C17:0)/(iso−C15:0)	0.20	0.29	0.02
*Ruminococcus albus*	Y=6.87−0.007/(C11:0)	0.26	0.18	0.03
*Ruminococcus flavafaciens*	Y=7.04−0.01/(C11:0)+35.41×C11:0×anteiso−C15:0	0.24	0.41	0.002
*Butyrivibro flarisolvens*	Y=7.30+73.80×C11:0×iso−C15:0+0.53×anteiso−C17:0×C17:0	0.12	0.38	0.09
*Eubacterium ruminantium*	Y=5.86−0.22/(C11:0)	0.28	0.15	0.04
*Streptococcus bovis*	Y=7.08−0.22/C17:0 +1.20×C11:0×anteiso−C17:0+0.65×C15:0×anteiso−C17:0	0.14	0.53	0.0005

R^2^ = coefficient of determination; RMSE = root mean square error.

**Table 11:** Predicted equations of ruminal bacterial bases from milk OBCFA.

Variables	Predicted Equation	R^2^	RMSE	*P*
Ruminal bases (g/kg DM)				
Cytosine	Y=1.54−0.79×C11:0×C17:0−0.93×iso−C17:0×anteiso−C17:0	0.32	0.09	0.01
Uracil	Y=1.18−4.11×iso−C15:0×iso−C17:0+1.59×anteiso−C15:0×iso−C16:0	0.50	0.10	0.0002
Guanine	Y=2.23+0.01/(C11:0)+1.17×C11:0×anteiso−C17:0+23.00×C13:0×anteiso−C17:0−1.48×iso−C17:0×anteiso−C17:0	0.58	0.20	0.001
Adenine	Y=1.96+2.33×iso−C16:0×iso−C16:0+52.67×C11:0×anteiso−C15:0−91.61×C13:0×iso−C16:0−0.5×anteiso−C17:0×C17:0	0.49	0.12	0.004
Ruminal bases /N (g/100 g N)				
Cytosine:N	Y=1.72+0.01/(C13:0)+0.20/(iso−C16:0)−1.90×iso−C16:0×anteiso−C17:0	0.58	0.18	0.002
Uracil:N	Y=2.93+0.01/(C13:0)−2.88×iso−C17:0×anteiso−C17:0+273.13×C11:0×C13:0−1.44×(iso−C15:0)/(iso−C17:0)	0.60	0.23	0.0004
Guanine:N	Y=7.17−2.12×iso−C17:0×anteiso−C17:0	0.37	0.50	0.001
Adenine:N	Y=3.27−2.53×iso−C17:0×C17:0+3.81×C15:0×iso−C16:0+0.73×(anteiso−C15:0)/(iso−C16:0)	0.62	0.28	<0.0001

N, the total contents of nitrogen in rumen. R^2^ = coefficient of determination; RMSE = root mean square error.
